# Neuroinflammation: From Molecular Basis to Therapy

**DOI:** 10.3390/ijms25115973

**Published:** 2024-05-29

**Authors:** Isabella Zanella

**Affiliations:** 1 Department of Molecular and Translational Medicine, University of Brescia, 25123 Brescia, Italy; isabella.zanella@unibs.it; Tel.: +39-030-3996806; 2 Cytogenetics and Molecular Genetics Laboratory, Diagnostic Department, ASST Spedali Civili di Brescia, 25123 Brescia, Italy

Neuroinflammatory conditions in the central nervous system (CNS) are implicated in the pathogenesis of several neuroimmune disorders such as acquired demyelinating syndromes, autoimmune encephalopathies, acute or chronic bacterial and viral CNS infections as well as multiple sclerosis (MS). Neuroinflammation also plays a crucial role in several neurodegenerative disorders [1]. Furthermore, the involvement of pathogenic mutations in genes related to neuroinflammation in neurodegenerative conditions such as Parkinson’s disease (PD), amyotrophic lateral sclerosis (ALS), or different forms of dementia such as Alzheimer’s disease (AD) or frontotemporal dementia (FTD) is now ascertained [2]. 

The Special Issue “Neuroinflammation: From Molecular Basis to Therapy” brings together five original research articles and two review articles, highlighting the involvement of neuroinflammation in different pathological conditions.

The original research contribution by Fu and colleagues focuses on the protective effect of torpor-like hypothermia induced by the A1 adenosine receptor (A1AR) agonist N62-cyclohexyladenosine (CHA) against neuroinflammation. Induced hypothermia has been found to protect against brain damage caused by cerebral ischemia [3]. The authors explored the possible therapeutic effect of hypothermia in infectious diseases of the CNS and established a murine model of neuroinflammation via the intraperitoneal injection of the mice with lipopolysaccharide (LPS). They found that CHA co-treatment attenuated both apoptotic processes and inflammatory responses induced in the brain by LPS injection, also maintaining neuron morphology and reducing microgliosis. Mechanistically, CHA did not directly affect the inflammatory and oxidative-stress response of microglia, macrophages, or brain vascular endothelial cells to LPS, acting through A1A receptors; instead, the authors found, intriguingly, that the protective effect exerted by CHA on neuroinflammation was due to the activation of the thermoregulatory neurocircuitry that induced hypothermia and prevented blood–brain barrier (BBB) disruption. 

Although the highly effective combined antiretroviral therapy (cART) is currently used to treat HIV infection, mild forms of HIV-associated neurocognitive disorders (HANDs) still occur in infected and treated individuals. HIV invades the brain during the initial days following infection and induces a neuroinflammation state through the production of soluble pro-inflammatory mediators released by the infected cells and the neurotoxic effects of viral proteins on neurons. The chronic persistence of a neuroinflammation state may result in neurocognitive disorders [4]. Moreover, cART itself has the potential to induce neuroinflammation [5,6]. At present, the HIV integrase strand transfer inhibitors (INSTIs) are the first-line treatment regimens for the treatment of HIV infection [7]. More recently, the developmental safety of INSTIs has been discussed in several published works [8] and one of these drugs, in particular, dolutegravir, has been implicated in adverse fetal development, neuroinflammation, and neuronal impairment in mice [9]. Among the INSTI class drugs, cabotegravir is the most recently approved drug for clinical use in HIV-infected patients. As part of this Special Issue, the study by Zizioli et al. explores the impact on neurodevelopment of exposure to cabotegravir, using zebrafish (*Danio rerio*) embryos as an in vivo model. Although exposure to therapeutic doses of the drug did not reduce the survival rate nor induce gross morphological malformations both in the brain region and the entire body during the developmental stages of embryos, the authors showed that cabotegravir exposure resulted in reduced larvae locomotion and a potential neurotoxic effect, acting on the modulation of transcription factors that are crucial for neurodevelopment.

The third original contribution included in this Special Issue by So and colleagues explores the role of the microglial fatty-acid-binding protein 4/uncoupling protein 2 (FABP4-UCP2) axis in the induction of cognitive decline in mice maintained on a high-fat diet (HFD) for 12 weeks. FABP4 is a protein belonging to the family of fatty acid-binding proteins (FABPs), involved in fatty acid metabolism and trafficking. FABP4 is prevalently expressed in white and brown adipocytes, monocytes, macrophages, and endothelial cells, where it plays a central role in not only lipolysis and the transport and storage of lipids but also adipose tissue and vascular inflammation [10]. The authors of a recent study identified an additional role of FABP4 in neuroinflammation [11]. A multitude of experimental data show that an HFD may induce metabolic syndrome, accompanied by microglial activation and neuroinflammation [12]. Through the use of a murine model lacking the FABP4 gene, the authors demonstrated that while HFD-fed wild-type mice exhibited impaired memory suggestive of cognitive decline, increased hippocampal expression of inflammatory cytokines, and increased expression of ionized calcium-binding adaptor molecule 1 (IBA1), suggestive of microglia activation and reduced microglial UCP2 expression, the loss of FABP4 in the knock-out mice reversed all of the effects caused by HFD exposure. 

Trimethyltin (TMT) is an endocrine-disrupting chemical belonging to the organotin compound family that was used in the past as a pesticide and wood preservative; however, its use was restricted in many countries due to its neurotoxic effects. Nonetheless, TMT still persists in the environment, and TMT exposure may occur following the intake of contaminated food and water. TMT neurotoxicity after exposure may result in memory loss, in addition to several other neurologic manifestations such as hyperactivity, seizures, and ataxia, among others. TMT neurotoxicity has been linked to several potential mechanisms, such as the disruption of neuroendocrine pathways or the induction of oxidative stress, mitochondrial damage, and pro-inflammatory pathways [13]. The authors of the fourth original contribution included in this Special Issue propose hydrogen gas (H_2_) inhalation to ameliorate cognitive impairment induced by TMT exposure in a murine model. The authors demonstrate that H_2_ inhalation reduced the occurrence of seizures and cognitive dysfunction, demonstrating the protective effect of inhaled H_2_ derived from the reduction in reactive oxygen species, nitric oxide, Ca^++^, and malondialdehyde levels in the serum and brains of TMT-treated mice. The authors further showed that TMT exposure was accompanied by an increase in antioxidative enzyme activity and the induction of pro-inflammatory cytokines in the brain, while H_2_ inhalation was found to restore the mice’s physiological levels. Moreover, the authors showed that H_2_ inhalation decreased TMT-induced levels of AD-related biomarkers (Aβ-40, phospho-tau, and Aβ aggregates) and attenuated TMT-induced apoptosis in the brain. 

Mutations in the genes encoding for alpha-synuclein (α-syn) and leucine-rich repeat kinase 2 (LRRK2) are both implicated in PD. α-syn mutations (duplication or triplication of the gene and point missense mutations) are associated with early-onset PD, characterized by the aggregation of α-syn and other proteins in the Lewy bodies (LBs). Mutations in LRRK2 also induce the presence of LBs in which the LRRK2 protein has been found in some cases. LRRK2 mutations often act through the increase in LRRK2 kinase activity, related to the C-terminal part of LRRK2. Evidence from a number of studies suggests that the two proteins, α-syn and LRRK2, may functionally interact, and thus, these proteins may both contribute to determining disease progression [14]. The authors of the final original contribution included in this Special Issue explore the potential synergistic effect of two pathogenic mutations, one in the α-syn gene (A53T) and the second in the C-terminal domain of the LRRK2 gene (G2091S), considering potential therapeutic implications (i.e., the use of LRRK2 kinase activity inhibitors). Cresto and colleagues studied this possible interplay, using adult rats transfected with the C-terminal domain of wild-type G2091S or G2091S/D1994A (dead kinase, DK, form) mutants of LRRK2, alone or in combination with wild-type or A53T mutated α-syn and expressing the wild-type and/or mutated proteins in dopaminergic (DA) neurons. The authors interestingly found higher DA neuron degeneration induced by the combination of the two A53T α-syn and G2091S LRRK2 mutated proteins in comparison with the two mutations alone, suggesting a potentiated neurotoxic and deleterious effect of A53T mutated α-syn through the presence of the G2091S mutated kinase domain of LRRK2, further highlighting the potential therapeutic implications of the use of LRRK2 kinase activity inhibitors.

In their review article, Bikbova and colleagues focused their attention on inflammation in diabetes, particularly in one complication of diabetes, that is, neuropathy of the retina. Previously considered a microvascular complication of diabetes, diabetic retinopathy is now defined as a neurodegenerative process that precedes microvascular abnormalities of the retina [15]. The authors reviewed literature evidence regarding the presence of several pro-inflammatory molecules in the ocular fluid of diabetic patients and their role in the early death of retinal neurons and inducing vascular dysfunctions. In turn, the resident retinal microglia become activated and produce further pro-inflammatory mediators that exacerbate the impaired functionality of the retinal microvasculature. Finally, they focused on the treatment of diabetic retinopathy, reviewing the most recent and advanced approaches—from the use of new anti-inflammatory and anti-hyperlipidemic therapies to the use of drugs that act in the prevention of neuronal cell death.

In the last contribution included in this Special Issue, a second review article, Figarola-Centurion and colleagues propose the use of sirtuin (SIRT) modulators to improve cognitive decline in people living with HIV (PLWH). The authors first reviewed the role of the trans-activator of transcription (Tat) protein of HIV in HANDs. Tat is released by infected cells in the extracellular matrix and can enter neurons via endocytosis. Tat can alter these cells via several mechanisms, such as disruption of mitochondrial fusion–fission dynamics and mitophagy or the induction of apoptosis. Tat also interacts with sirtuins, in particular SIRT1, 2, and 3. SIRT1 is required for the Tat Lys50 deacetylation needed for Tat-trans-acting responsive element (TAR) complex formation; Tat also inhibits SIRT1 activity, and this inhibition may in turn cause neuronal loss. Tat also physically interacts with SIRT2. This sirtuin plays a central role in mitochondrion homeostasis; the authors hypothesized that Tat-induced mitochondrial dysfunctions may be related to this Tat/SIRT2 interaction. Finally, the mitochondrial SIRT3 is strictly involved in the antioxidant response, biogenesis, and DNA integrity of the mitochondrion and the mitophagy pathway. Tat downregulates the expression of SIRT3 in the microglia, modulating the antioxidant response and release of pro-inflammatory cytokines. The authors conclude their study with a discussion on the possible modulation of SIRT1, 2, and 3 through the use of several drugs and natural compounds as adjuvant treatment in HIV infection in order to prevent the development of HANDs.

In conclusion, the following Special Issue highlights new evidence of the central role of neuroinflammation in several pathological conditions, from neurodegenerative diseases to infectious and metabolic diseases and conditions induced by exposure to neurotoxicants. The authors of the aforementioned contributions also suggest interesting therapeutic approaches and strategies to treat and improve the outcomes of the above pathological conditions.
